# MiR-181b serves as diagnosis and prognosis biomarker in severe community-acquired pneumonia

**DOI:** 10.1590/1678-4685-GMB-2020-0431

**Published:** 2021-08-27

**Authors:** Qiaolian Li, Tingting Wu, Song Li

**Affiliations:** 1 Shanxian Dongda Hospital, Department of Respiratory and Critical Care Medicine, Heze, China.

**Keywords:** Severe community-acquired pneumonia, miR-181b, diagnosis, prognosis

## Abstract

Severe community-acquired pneumonia (SCAP) is a common critical disease in the intensive care unit (ICU). This study aims to evaluate the clinical significance of miR-181b in SCAP, which has been revealed to be dysregulated in acute respiratory distress syndrome events due to SCAP. There were 50 SCAP patients and 26 healthy volunteers were recruited in this study. The expression of miR-181b was detected by RT-qPCR and the difference between SCAP and healthy controls was evaluated. The diagnosis and prognosis value of miR-181b was assessed by the receiver operating characteristics (ROC), Kaplan-Meier, and Cox regression analysis. miR-181b was significantly downregulated in SCAP compared with healthy controls. The downregulation of miR-181b showed a significant association with the white blood cell count, absolute neutrophils, and the C-reactive protein of patients. The downregulation of miR-181b could distinguish SCAP patients from healthy controls and predicate the poor prognosis of SCAP patients. Downregulated miR-181b serves as a diagnosis and prognosis biomarker for SCAP, which may be useful biological information for the early detection and risk estimation of SCAP.

## Introduction

Pneumonia is one of the lower respiratory tract infections, which accounts for the high mortality of patients (Robert et al., 2018). Severe community-acquired pneumonia (SCAP) is the most frequent and severe type of pneumonia, requiring hospitalization and intensive care unit (ICU) treatment with the high mortality rates of 30-50% in ICU (Jain et al., 2015a; Torres et al., 2019). Despite the occurrence of SCAP has been decreased in the past decades, SCAP remains a challenge in the clinic (Murdoch, 2016). The proportion of primary viral pneumonia among all causes of SCAP is underestimated, which is comparable to the proportion of bacterial pneumonia (Jain *et al*., 2015b). Due to the limitations in the detection of specific pathogen responsible for SCAP and the lack of clinical guidelines, the early detection and the prognosis of SCAP patients were still unsatisfactory (Murdoch *et al*., 2009). Identification of potential biomarkers could improve the clinical care of patients and provide novel therapeutic strategies. 

MicroRNAs (miRNAs) are highly conserved composed of 18-25 nucleotides, which have been demonstrated to play vital roles in the posttranscriptional regulation of gene expression in the pathogenesis of lung disease and infections (Cao et al., 2016). Previously, the combined expression of miR-126, miR-27a, miR-146a, and miR-155 was revealed to predict acute respiratory distress syndrome, which is the most frequent complication of CAP (Wu et al., 2019). miR-29c was found to be negatively associated with the IgG, IgM level of *Mycoplasma pneumoniae*, and via targeting B7-H3, miR-29c exerted inflammatory immune response to *M. pneumoniae* infection (Li et al., 2019a). miR-181b was revealed to be downregulated in the serum of CAP patients with acute respiratory distress syndrome in the previous study. The functional role of miR-181b has been reported in various diseases, such as pulmonary arterial hypertension, non-small lung cancer, and many other cancers (Liu et al., 2018; Zhou et al., 2019; Zhao et al., 2020). It was speculated that the downregulation of miR-181b might imply the clinical value in the diagnosis and prognosis of SCAP. 

The purpose of this study is to estimate the clinical significance of miR-181 in SCAP and confirm whether miR-181 could be used as diagnosis and prognosis biomarkers for the discrimination of SCAP and the prediction of SCAP development.

## Material and Methods

### Subject recruitment and sample collection

A total of 50 SCAP patients were enrolled from the ICU of Shanxian Dongda Hospital during 2017-2019. Another 26 healthy volunteers that had normal physical examinations were recruited as the control group. The diagnosis of SCAP patients was based on the presence of pulmonary infiltrates on the chest and the clinical presentation, including cough, sputum production, dyspnea, fever > 37.8 °C. The severity was evaluated by the pneumonia severity index (PSI). Pneumonia developing during hospitalization was excluded. This study was approved by the Ethics Committee of Shanxian Dongda Hospital (No. 2016045) and informed consent was obtained from each participant or their guardians. A 28-day follow-up survey was conducted to obtain the survival information of all participants. Serum samples were collected within 24 h of ICU admission and stored at -80 °C for further analysis.

### Biochemical measurements

The serum concentration of C-reactive protein was measured by the turbidimetric inhibition immune assay. The lactate dehydrogenase was determined according to the previous study (Sikkink and Ramirez-Alvarado, 2010). The absolute neutrophils and white blood cell count were analyzed using an automated hematology analyzer (Sysmex XE-2100, Sysmex, Japan).

### Real-time quantitative PCR (RT-qPCR)

Total RNA was extracted with TRIzol reagent (Invitrogen, Carlsbad, CA, USA). Extracted RNA was transcribed reversely to cDNA was generated by the PrimeScript RT reagent Kit (Takara, Tokyo, Japan). The expression of miR-181b was analyzed by the Applied Biosystems 7900 Real-Time PCR system (Applied Biosystems, Foster City, CA) with the SYBR Green I Master Mix Kit (Invitrogen, Carlsbad, CA, USA). The 2^−ΔΔCt^ method was used to calculate the relative expression level of miR-181b with U6 as the internal standard. The primer sequences used were as follows: miR-181b forward 5’-GCGGATCATTCATTGCTGTCG-3’, reverse 5’-GTGCAGGGTCCGAGGT-3’; U6 forward 5’-GACCTCTATGCCCAACACAGT-3’, reverse 5’-AGTACTTGCGCTCAGGAGGA-3’.

### Statistical analysis

Data were represented as mean ± SD. and analyzed by SPSS version 23.0 software (SPSS Inc., Chicago, IL) and GraphPad Prism 7.0 software (GraphPad Software, Inc., USA). Differences between groups were assessed by student’s t-test or one-way ANOVA. The association between miR-181b expression level and the clinical characteristics of patients was estimated by the χ^2^ test and Pearson’s correlation analysis. Kaplan-Meier analysis and Cox regression analysis were employed to estimate the prognostic value of miR-181b in SCAP. The diagnostic value of miR-181b was evaluated by the receiver operating characteristic (ROC) and the values of area under the curve (AUC) with 95% confidence interval (95% CI) were also calculated. It was statistically significant when *P* < 0.05.

## Results

### Clinical characteristics of SCAP patients and healthy volunteers

As shown in Table 1, the recruited SCAP patients include 29 males and 21 females with PSI scores of 110.29 ± 23.50 years old and an average age of 7.30 ± 0.42 years old. The age and the sex of SCAP patients and healthy volunteers showed no significant difference (*P* > 0.05). While, the white blood cell count (*P* = 0.003), absolute neutrophils (*P* < 0.001), and the concentration of C-reactive protein (*P* < 0.001) of SCAP patients were significantly higher than those of healthy volunteers.

**Table 1 -:** Clinical characteristics of SCAP patients and healthy volunteers.

Parameters	Healthy control (n = 26)	SCAP (n = 50)	*P* Value
Age (years)	6.81 ± 0.43	7.30 ± 0.42	0.778
Sex (male, %)	16, 61.54%	29, 58.00%	0.540
White blood cell count (x 10^9^/L)	5.91 ± 1.72	8.85 ± 1.22	0.003
Absolute neutrophils (x 10^9^/L)	3.94 ± 1.69	43.83 ±10.84	< 0.001
C-reactive protein (mg/L)	0.23 ± 0.06	11.73 ± 4.94	< 0.001
Lactate dehydrogenase (U/L)	231.06 ±52.69	388.18 ± 45.69	0.376

### The serum expression level of miR-181b in SCAP patients and its association with the clinical characteristics of patients

**Figure 1 -:**
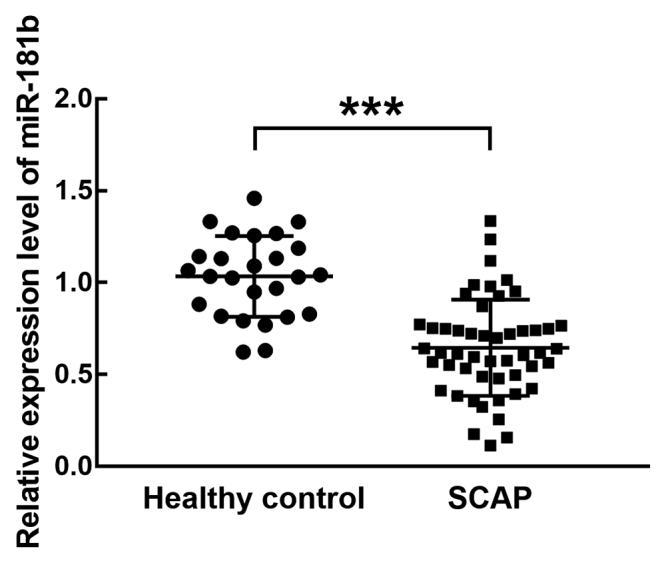
Expression level of miR-181b in SCAP and healthy volunteers. miR-181b was significantly downregulated in SCAP compared with healthy controls. The data were shown as mean ± SD. and analyzed by unpaired Student’s *t*-test. *P* < 0.001.

The serum expression level of miR-181b was significantly lower in SCAP patients compared with healthy controls (*P* < 0.001, Figure 1). According to the average expression level of miR-181b in SCAP patients, the SCAP patients were divided into the high miR-181b group (n = 21) and the low miR-181b group (n = 29). The association between the miR-181b expression level and the clinical characteristics of SCAP patients was evaluated. Results showed that the expression of miR-181b was significantly associated with the white blood cell count (*P* = 0.018), absolute neutrophils (*P* = 0.035), the concentration of C-reactive protein (*P* = 0.040), and the PSI scores (*P* = 0.021) of patients (Table 2). While the age, sex, and lactate dehydrogenase showed no significant association with the expression level of miR-181b (*P* > 0.05, Table 2).

**Figure 2 -:**
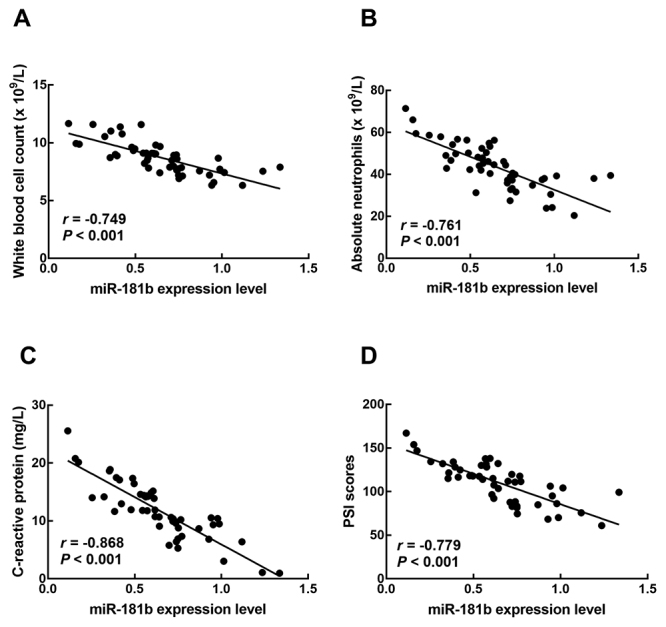
Correlation between miR-181b expression level and white blood cell count (A), absolute neutrophils (B), C-reactive protein concentration (C), and PSI scores (D) of SCAP patients.

Additionally, the significant positive correlations between the miR-181b expression level and the white blood cell count (*r* = -0.749, *P* < 0.001), absolute neutrophils (*r* = -0.761, *P* < 0.001), c-reactive protein concentration (*r* = -0.868, *P* < 0.001), and the PSI scores (*r* = -0.779, *P* < 0.001) were also validated by the Pearson’s correlation analysis (Figure 2).

**Table 2:** Association between miR-181b expression level and the clinical characteristics of SCAP patients.

Parameters	Total patients (n = 50)	miR-181b expression leve		*P* Value
		High miR-181b (n = 21)	Low miR-181b (n = 29)	
Age				0.851
< 7	15	6	9	
≥ 7	35	15	20	
Sex				0.634
Male	29	13	16	
Female	21	8	13	
White blood cell count (x 10^9^/L)				0.018
< 8	19	12	7	
≥ 8	31	9	22	
Absolute neutrophils (x 10^9^/L)				0.035
< 40	20	12	8	
≥ 40	30	9	21	
C-reactive protein (mg/L)				0.040
< 10	18	11	7	
≥ 10	32	10	22	
Lactate dehydrogenase (U/L)				0.094
< 380	24	13	11	
≥ 380	26	8	18	
PSI score				0.021
< 90	15	10	6	
≥ 90	35	11	24

### The diagnostic value of miR-181b in SCAP

**Figure 3 -:**
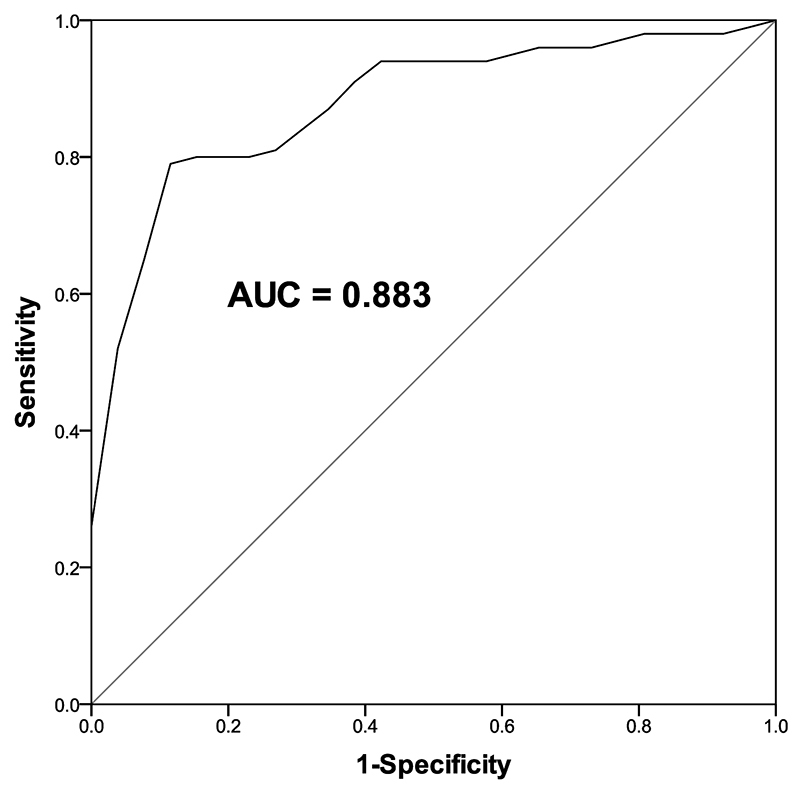
ROC curve analysis of miR-181b for discriminating SCAP patients from healthy controls. The AUC of the ROC curve is 0.883, the sensitivity and specificity are 0.780 and 0.923, respectively.

The diagnostic value of miR-181b in SCAP was assessed with the employment of the receiver operating characteristics (ROC) curve. The ROC curve showed that miR-181b could discriminate SCAP patients from healthy volunteers with the area under the curve of 0.883, and the sensitivity and specificity of 0.780 and 0.923, respectively (Figure 3).

### The prognostic value of miR-181b in SCAP

**Figure 4 -:**
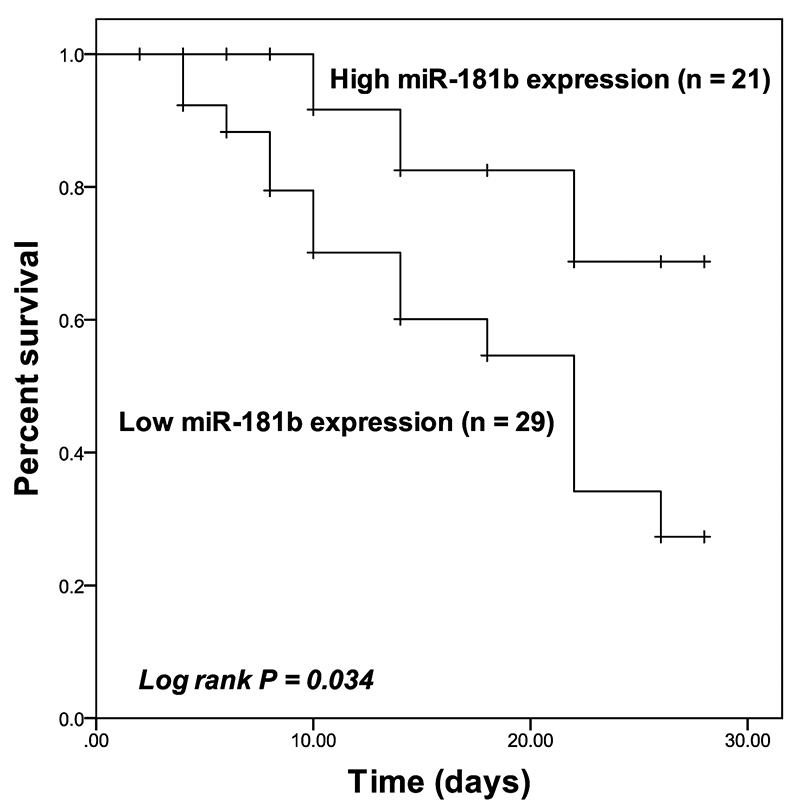
Kaplan-Meier curve of SCAP patients with different expression of miR-181b. Patients with high miR-181b expression had a significantly higher survival rate than that of patients with low miR-181b expression. Log-rank *P* = 0.034.

The survival rate of SCAP patients was plotted as the Kaplan-Meier curve shown in Figure 4. Patients in the high miR-181b group showed a better survival rate than that of patients in the low miR-181b group and the difference was significant (Log rank *P* = 0.034). Additionally, the results of Cox regression analysis showed that miR-181b (HR value = 6.932, 95% CI = 1.471-32.668, *P* = 0.014) and the PSI scores (HR value = 5.652, 95% CI = 1.281-24.936, *P* = 0.022) served as independent indicators for the prognosis of SCAP patients (Table 3).

**Table 3 -:** Correlation between clinical parameters and survival of SCAP patients by Cox regression analysis.

Parameters	HR value	95% CI	*P* value
miR-181b	6.932	1.471-32.668	0.014
Age	1.252	0.291-5.395	0.763
Sex	1.512	0.408-5.601	0.536
White blood cell count	1.884	0.492-7.212	0.355
Absolute neutrophils	0.683	0.171-2.734	0.590
C-reactive protein	2.763	0.594-12.852	0.195
Lactate dehydrogenase	1.643	0.502-5.378	0.412
PSI scores	5.652	1.281-24.936	0.022

## Discussion

SCAP is one of the most common critical diseases in pediatric ICU (PICU), which always developed from mild pneumonia with a high incidence rate (Hon et al., 2015). Nowadays, the clinical diagnosis and prediction of SCAP depend on some regular analyses, which lack specificity and are always diagnosed at an advanced stage (Qi et al., 2015). Therefore, the novel diagnosis method and the prognosis prediction with high sensitivity and specificity are necessary for SCAP. Currently, the clinical significance of miRNAs, a series of non-coding RNAs with a length of 18-25 nucleotides, has drawn special attention (Bartel 2004). A number of miRNAs have been revealed to serve as diagnosis and prognosis biomarkers in cancers, cardiovascular diseases, and neurological diseases (Wang et al., 2016; Li et al., 2019b). For instance, cardiac aging induced overexpression of miR-34a, miR-34a regulated cardiac contractile function during aging by targeting PNUTS (Boon et al., 2013). In aneurysmal subarachnoid hemorrhage, miR-1297 acts as an independent predictive factor of the outcome at 1 year of patients (Sheng et al., 2018). While there are few studies on the identification of novel biomarkers for SCAP, which limited the treatment and the management of SCAP.

The major finding of the present study revealed that miR-181b was downregulated in SCAP compared with healthy controls, and showed significant association with the white blood cell count, absolute neutrophils, C-reactive protein concentration, and PSI scores of SCAP patients, which are important clinical and laboratory characteristics of patients (Mukamal et al., 2010; Rhim et al., 2011). miR-181b was previously reported to be downregulated in SCAP patients with acute respiratory distress syndrome and demonstrated to play role in various human diseases, such as ischemic stroke, Parkinson’s disease, and coronary artery disease (Guo et al., 2018; Han et al., 2018; Li et al., 2018). There are also several miRNAs reported to be dysregulated and mediate the development of pneumonia. For example, miR-222-3p was upregulated in the peripheral blood plasma of pneumonia children, especially those with pleural effusion (Chu et al., 2019). miR-146b could alleviate inflammation injury in pediatric pneumonia via inhibiting MyD88/NF-κB signaling pathway (Zhang et al., 2020). The abnormal expression of miR-181b implied the potential function of miR-181b in SCAP. The white blood cell count and C-reactive protein are useful for predicting clinical outcomes of children hospitalized with CAP and associated with the fever duration and hospital length of stay (Williams et al., 2015). Therefore, miR-181b was speculated to be associated with the occurrence and development of SCAP.

Previously, miR-181b was identified as biomarkers in a variety of diseases. In colorectal cancer and acute myeloid leukemia, miR-181 could predict the poor survival of patients (Guo et al., 2017; Peng et al., 2019). miR-181 was demonstrated to be associated with the lymph-node metastasis of oral squamous cell carcinoma and to serves as a marker for screening osteoarthritis patients (Xia et al., 2017; Yang et al., 2011). Here, the downregulation of miR-181b was found to differentiate SCAP from healthy volunteers indicating the diagnosis biomarker role of miR-181b in SCAP. The survival of SCAP patients was positively correlated with the expression level of miR-181b and miR-181b acts as an independent indicator of the prognosis of SCAP patients.

There are several minor limitations to this study. Due to the limitation of recruited patients, the sample size of this study was small and only a severe population was included. Likely, the results of the present study may not be generalizable to a non-selective population of patients with pneumonia. In previous studies that focused on the function of miR-181b in other human diseases, TGF-β was identified as a direct target of miR-181b (Hori et al., 2017; Yao et al., 2018). Further, the association between miR-181b and inflammatory response was also considered as a vital pathway that made miR-181b be involved in the disease development (Wang et al., 2019; Zhao et al., 2020). These potential mechanisms underlying the function of miR-181b in SCAP need further experiments and validations. However, this study still provides a clinical reference for the management of SCAP patients. 

Taken together, miR-181b was downregulated in SCAP and associated with the white blood cell count, absolute neutrophils, C-reactive protein concentration, and the PSI scores of SCAP patients. The downregulation of miR-181b could serve as diagnosis and prognosis biomarkers for the early screening and outcome prediction of SCAP patients.
